# Evaluating Water Level Changes at Different Tidal Phases Using UAV Photogrammetry and GNSS Vertical Data

**DOI:** 10.3390/s19173778

**Published:** 2019-08-31

**Authors:** Norhafizi Mohamad, Mohd Faisal Abdul Khanan, Anuar Ahmad, Ami Hassan Md Din, Himan Shahabi

**Affiliations:** 1 Department of Geoinformation, Faculty of Built Environment and Surveying, Universiti Teknologi Malaysia (UTM), Johor Bahru 81310, Malaysia; 2 Geomatics Innovation Research Group, Faculty of Built Environment and Surveying, Universiti Teknologi Malaysia (UTM), Johor Bahru 81310, Malaysia; 3 Department of Geomorphology, Faculty of Natural Resources, University of Kurdistan, Sanandaj 66177-15175, Iran

**Keywords:** water level changes, UAV photogrammetry, tidal phase, GNSS, Kilim River

## Abstract

Evaluating water level changes at intertidal zones is complicated because of dynamic tidal inundation. However, water level changes during different tidal phases could be evaluated using a digital surface model (DSM) captured by unmanned aerial vehicle (UAV) with higher vertical accuracy provided by a Global Navigation Satellite System (GNSS). Image acquisition using a multirotor UAV and vertical data collection from GNSS survey were conducted at Kilim River, Langkawi Island, Kedah, Malaysia during two different tidal phases, at high and low tides. Using the Structure from Motion (SFM) algorithm, a DSM and orthomosaics were produced as the main sources of data analysis. GNSS provided horizontal and vertical geo-referencing for both the DSM and orthomosaics during post-processing after field observation at the study area. The DSM vertical accuracy against the tidal data from a tide gauge was about 12.6 cm (0.126 m) for high tide and 34.5 cm (0.345 m) for low tide. Hence, the vertical accuracy of the DSM height is still within a tolerance of ±0.5 m (with GNSS positioning data). These results open new opportunities to explore more validation methods for water level changes using various aerial platforms besides Light Detection and Ranging (LiDAR) and tidal data in the future.

## 1. Introduction

Exposure to tidal influence causes some rivers to have similar characteristics to coastal zones that is, they experience tidal inundations every 24 hours. One method used to expand our knowledge about tidal inundation in tidal rivers is field surveys using tide gauge instruments [1,2,3,4,5]. Tidal inundation evolves dynamically according to the alignment of the sun and moon, the pattern of tides in the deep ocean, and the shape of the coastline and near-shore bathymetry [6,7]. Hence, multi-source data with different epochs are required, and many solutions have been developed over the last decade. Measuring the water level using satellite images was discussed by [8,9,10,11] and was used to analyze water level changes at different tidal phases [12,13,14,15,16]. Instead of using Structure from Motion (SFM), digital surface models (DSMs) could be produced from high-resolution satellite images [17,18,19,20,21,22,23]. The other method to produce DSMs is through Light Detection and Ranging (LiDAR) and Terrestrial Laser Scanning (TLS), which allow the generation of accurate DSMs with comparable spatial resolution [24,25,26,27,28,29,30]. However, the availability of satellite images is limited because of uncertain weather, while the high cost of LiDAR and TLS acquisition limits the number of field measurements. 

To measure the water level at different tidal phases, an unmanned aerial vehicle (UAV) combined with a Global Navigation Satellite System (GNSS) appears to be an efficient solution. Several studies showed the performance of this technique in tidal and intertidal areas; the vertical accuracy of the DSM was about ±10 cm [31,32,33,34,35,36]. Although a UAV is affected by weather and meteorological conditions such as rain and strong wind, it is still able to collect images that allow the production of a three-dimensional (3D) point cloud and DSM using the SFM process. Bad weather and meteorological conditions could be avoided by planning data acquisition on a fine day and in good weather conditions by referring to the weather forecast. The accuracy of the DSM was already proved to be similar to that with LiDAR data [37]. In [38], the authors summarized several advantages of UAVs: (1) a high level of automation of photographic survey; (2) very low operating cost; (3) high repeatability of the survey; and (4) the possibility to get aerial photography with centimetric resolution [39]. In addition, UAVs are also used for a wide range of hydrology and hydraulic applications such as fluvial monitoring, erosion detection, river bathymetry, and geomorphology using photogrammetric techniques [40,41,42,43,44].

In this study, we demonstrate the evaluation of water level changes at different tidal phases using a DSM from UAV devices supported by high-accuracy positioning using a GNSS receiver. The DSM from UAV data provides water level measurement with higher accuracy as measured by its similarity to the water level from tide gauge measurement. Low-orbital flight with a GNSS positioning system is the best combination to measure water level changes in a small river and an inaccessible environment, such as in a mangrove forest. 

## 2. Study Area

**Figure 1:**
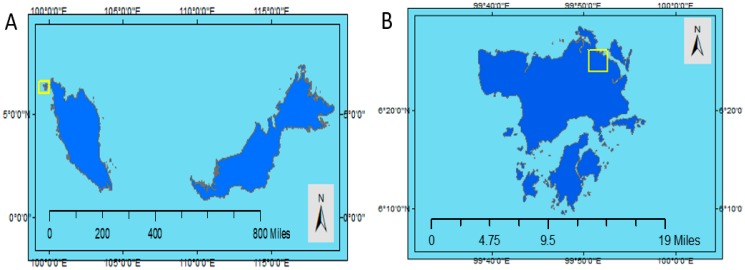

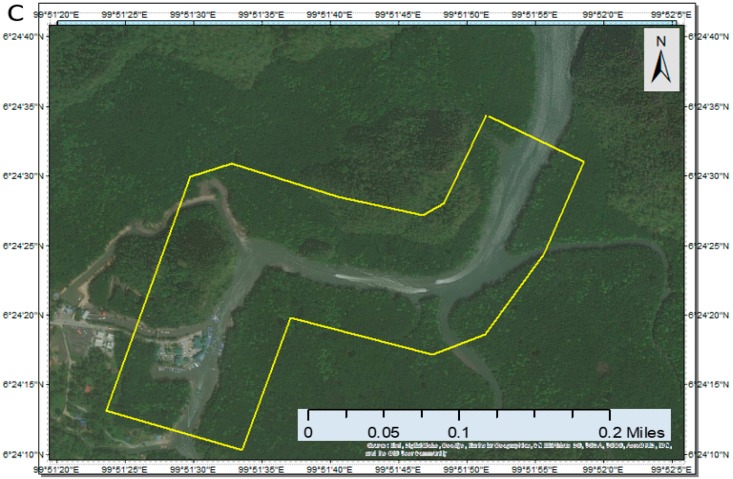
Location of the study area; (**A**) Location of the study area on a map of Peninsular Malaysia; (**B**) Location of the study area on Langkawi Island; (**C**) Location of the study area on the Kilim River.

The selected study area was at Kilim River, Langkawi Island, Kedah, Malaysia (Figure 1). This study area received recognition as the UNESCO Kilim Karst Geoforest Park (KKGP) in 2007. The Kilim River is located at 6°21.518′–6°26.093′ N and 99°51.159–99°51.159′ E, allowing this river to have a semi-diurnal tide, and this river also experiences two high and two low tides per day [45]. Although the Kilim River is approximately 3 kilometers from the coastal area, the tidal effect still exists and affects the river. During the tidal phenomenon, the water level increases as much as 12 hours and 25 minutes apart, and it takes 6 hours and 12.5 minutes to go from low to high tide and vice versa. The situation is similar to the coastal area but different in some aspects such as the narrow width of the channel, high slope of the riverbank, and thick mangrove forest along the river. During high tides, the impact of tidal inundation is obvious and visible because of the sinking of the riverbank, especially in the flat slope area.

## 3. Materials and Methods

### 3.1. Specifications of the UAV

**Figure 2:**
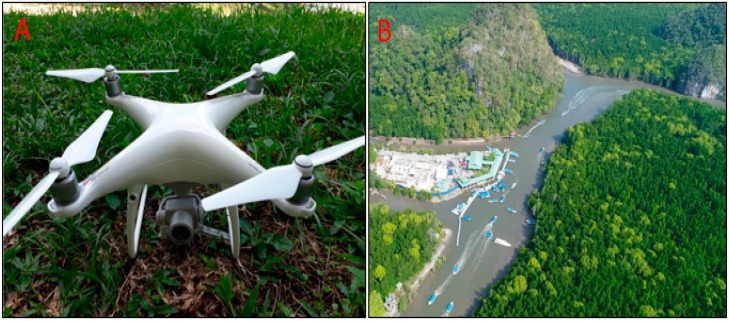
(**A**) The DJI Phantom 4 model used in this study; (**B**) An aerial view of study area captured by unmanned aerial vehicle (UAV).

The UAV equipment used in this study was a Da-Jiang Innovations (DJI) Phantom 4 Advanced model, developed by SZ DJI Technology Corporation Limited (Shenzhen, China). This model is of medium size, with a net weight of 1388 g and a diagonal wheelbase (propeller size excluded) of only 350 mm (Figure 2A). Figure 2B shows an example of an aerial image captured by a DJI Phantom 4 UAV. The DJI Phantom 4 Advanced is able to fly with a maximum altitude of 6000 m and a flying range of 5000 m. Equipped with a 5870 mAH LiPo 4S battery, the DJI Phantom 4 Advanced has approximately 30 minutes of maximum flight time, and its maximum wind speed resistance ranges from 29 to 38 kph. It is equipped with a remote controller with a 2.4–2.483 GHz operating frequency with a maximum transmission distance of 5 km according to the Federal Communication Commission (FCC) and 3.5 km according to Conformité Européenne (CE). The DJI GO 4 was used with a mobile app that allowed us to plan the flight before the mission and to interact with the UAV during the flight using a 2.4 GHz ISM line view working frequency. 

The UAV was mounted with a 1” Complementary Metal Oxide Semiconductor (CMOS) sensor with 20 megapixels. The DJI Phantom 4 Advanced has focal length 84° 8.8 mm/24 mm (35 mm format equivalent), an f/2.8-11 aperture with 1 m to ∞ (auto focus) shooting range, and shutter speed around 8-1/8000 s (electronic) and 8-1/2000 s (mechanical). An FC 6310 was attached to this UAV with focal length 8.8 mm, pixel size 2.61 × 2.61 µm, and resolution 4864 × 3648. The photography modes comprise single shot, burst shooting: 3/5/7 frames, auto exposure bracketing system (AEB): 3/5 bracketed frames at 0.7, exposure bias, time lapse, and High Dynamic Ranges (HDR) [46]. This model has ±0.1 m (with vision positioning) or ±0.5 m (with GPS positioning) vertical hover accuracy, while the horizontal accuracy is ±0.3 m (with vision positioning) or ±1.5 m (with GNSS positioning) [46].

### 3.2. Data Collection

#### 3.2.1. UAV Image Acquisition

**Figure 3:**
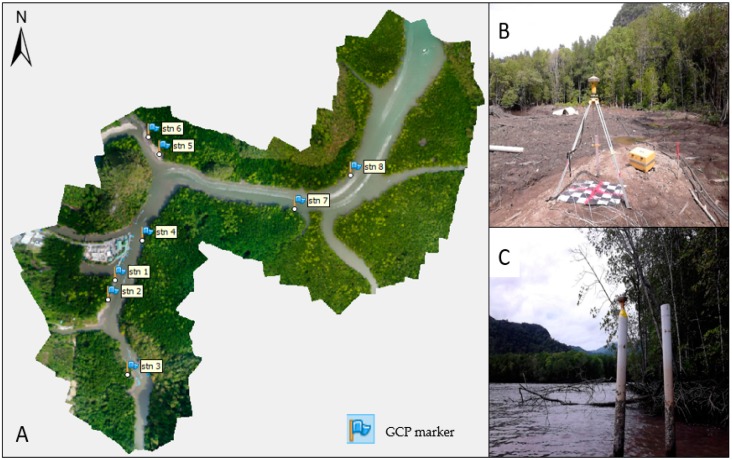
(**A**) Ground Control Points (GCP) distributions at the study area; (**B**) GCP 6 on the ground surface; (**C**) GCP 8 on the ground surface.

Two epochs of data collection were executed to identify the water level at different tidal phases of the Kilim River. Two flights were required to cover the whole study area for each epoch. Epoch 1 (20 December 2017) was collected during high tide, and Epoch 2 (20 December 2017) was collected during low tide. The average flying height for Epoch 1 was 184 m, while that for Epoch 2 was 228 m above ground level, which yielded images with a spatial resolution of 4.7 cm at Epoch 1 and 5.6 cm at Epoch 2. The flight plan was prepared using DJI GO 4 software, and the mission area was saved for each epoch. In total, 116 images were captured at Epoch 1 and 252 images were captured at Epoch 2 to cover the entire study area of about 0.688–0.689 km^2^, shown in Figure 3A.

The meteorological conditions and the tidal level were regular for both epochs. For the meteorological conditions, there was no strong wind or rain affecting the flight parameters (yaw, pitch, roll). However, the tidal range between both epochs was different since the tide was within a transition phase from high to lower tide conditions. At Epoch 1, the river was at low tide conditions, while at Epoch 2, the river was at high tide conditions (Table 1).

#### 3.2.2. GNSS Surveys

GNSS data are crucial to the image geo-referencing process and provide UAV products with high horizontal and vertical accuracy. Hence, ground control points (GCPs) were required for this study. Artificial targets (each a rubber mat with a highlighted “X” mark) were laid on the ground during the image acquisition process and were used later during image geo-referencing, as shown in Figure 3B. Eight GCPs were used since the study area was small, and two Topcon Trimble GR-5 models (Trimble Inc, Sunnyvale, CA, USA) were used as a GNSS receiver. The accuracy of the Topcon GR-5 model 3.0 mm + 0.5 ppm (horizontal) and 5.0 mm + 0.5 ppm (vertical), while that for Real-Time Kinematics (RTK) is 5 mm + 0.5 ppm (horizontal) and 10 mm + 0.8 ppm (vertical) [47]. Observation using a static technique was used within one hour for each station. The GPS observations used GDM 2000 as a local state coordinate system in the Kedah and Perak regions, and all stations were converted into the latitude and longitude format. As shown in Table 2, GPS stations were successfully measured at each location. The altitude of the ground surface is referred to as the ellipsoidal height and not the height above mean sea level (MSL).

**Figure 4:**
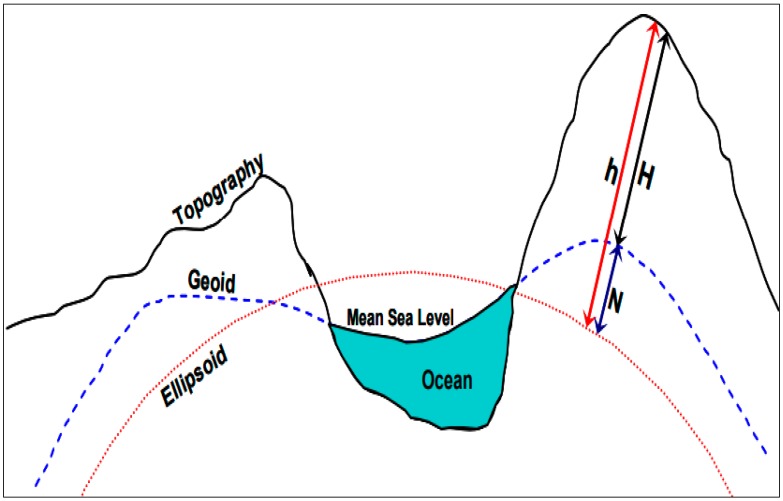
Relationship between the orthometric, ellipsoidal, and geoid heights [48].

To identify the height above MSL, three heights, which comprise the ellipsoidal, geoid, and orthometric heights, should be identified. The ellipsoidal height is the height derived from GNSS equipment, while the orthometric height is known as the height above mean sea level (Figure 4). The geoid model refers to gravity data which are collected through ground, airborne, or space gravity survey equipment. Once the geoid height is known, the orthometric height (H) can be measured based on the MyGeoid model, i.e., the Malaysian geoid model (Equation (1)). The geoid determination of Malaysia is based on gravimetry (airborne, surface, and satellite altimetry), which is located downward relative to the surface of the topography, after removal of a spherical harmonic reference field expansion [48,49]. The orthometric height is simplified as the difference between the geoid height and ellipsoidal height, as shown in Equation (1):

H = h − N,(1)

where

H = orthometric height;
h = ellipsoidal height;
N = geoid height.

### 3.3. Image Processing Using the SFM Algorithm

Measuring the water level at different tidal phases requires a riverbank 3D model. To generate a 3D model, running the images through a photogrammetric process called SFM is required. SFM reconstructs a relief from several stereoscopic images of the same object and reconstructs a 3D scene geometry from a set of images of a static scene by matching features on multiple images [50]. The SFM algorithm based on multi-views of the scene and the redundancy of the information allow the success of this process [51]. 

**Figure 5:**
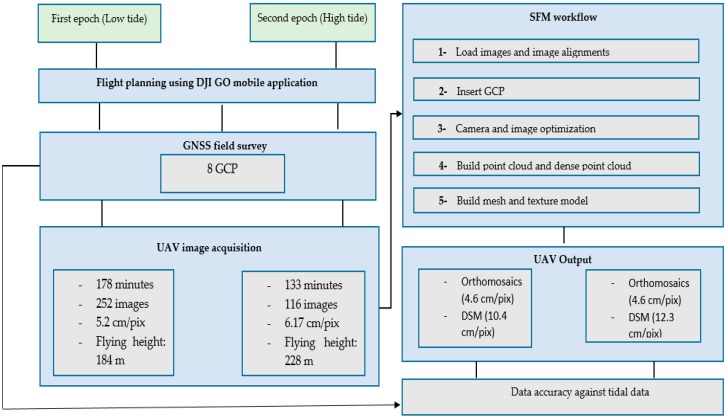
General overview of methods from flight planning to accuracy assessment of the digital surface model (DSM) with tidal data.

The SFM algorithm is available in several software products to generate DSMs and orthomosaics. Agisoft Photoscan® Professional Edition software (version 1.4.3, Agisoft LLC, St. Petersburg, Russia) was chosen as the processing software in this study. The workflow comprises several processes, including image alignment, camera calibration, camera optimization, point cloud and dense point cloud building, followed by the last step, which is mesh and model texture (Figure 5).

**Figure 6:**
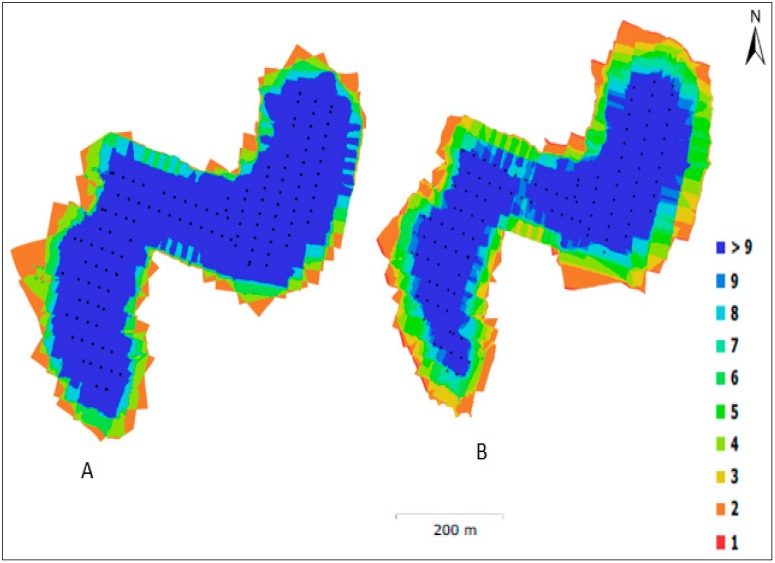
The camera locations and image overlap: (**A**) Epoch 1; (**B**) Epoch 2.

Generation of the DSM and orthomosaic image was started by aligning all aerial photos, followed by building a mesh (Figure 6). 

Later, the eight input marker coordinates containing GCP coordinates from GPS data were inserted manually (Table 2), and for altitude, the orthometric height was used instead of the ellipsoid or geoid height. Then, the process continued with optimizing the camera alignment so as to achieve higher accuracy in calculating the camera exterior orientation parameters and internal parameters and to correct the distortion. Subsequently, the aligned aerial photos were combined into a dense point cloud using the “build dense cloud” command. Afterward, the dense point clouds were generated using the “build mesh” command followed by “build texture” (with an optional polygonal model produced as a result). The final step in the Agisoft Photoscan software workflow included the “build DSM” process and the “build orthomosaic” command. The DSM and orthomosaic image were exported to an image file format such as JPEG, TIFF, or PNG for subsequent data processing.

### 3.4. DSM Generation

The dense point cloud was constructed at high quality and to an aggressive mode depth using Agisoft Photoscan software. These settings were time consuming since there were numerous tie points, especially in Epoch 2. Subsequently, the aligned aerial photos were combined into a dense point cloud using the “build dense cloud” command. Afterward, the dense point cloud was generated using the “build mesh” command followed by “build texture” (with an optional polygonal model produced as a result).

**Figure 7:**
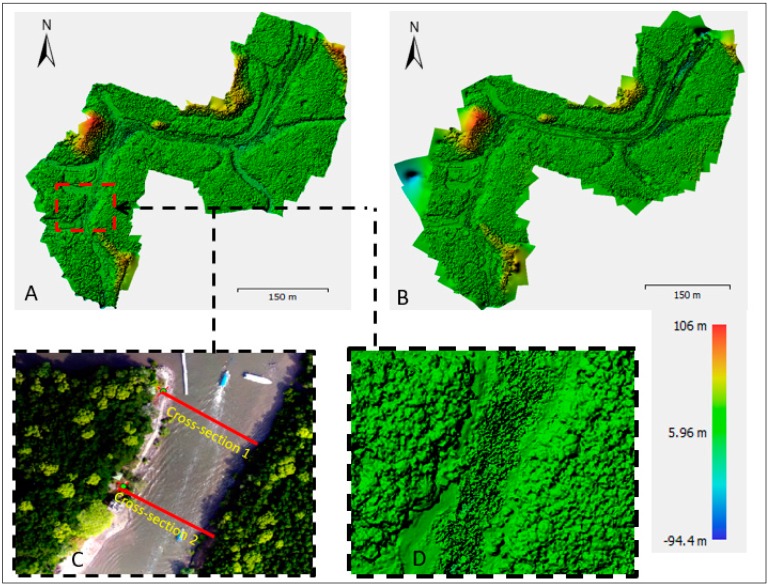
The DSM of the Kilim River; (**A**) At high tide (Epoch 1); (**B**) At low tide (Epoch 2); (**C**) Cross sections 1 and 2 in the orthomosaic image; (**D**) DSM at the area of cross sections 1 and 2.

The UAV data product comprises DSM and orthomosaic data types (Figure 7). Figure 7A displays the DSM of the Kilim River during high tide (Epoch 1), while Figure 7B displays the Kilim River during low tide (Epoch 2). Both the DSMs in Figure 7A,B show that the terrain condition ranges from 100 m to 90 m, specifically referring to the red area as hill and dark blue as the river surface. One focus area was selected for further analysis regarding water level changes during high and low tide. Two cross section lines were established in Figure 7C,D to mark the strategic area used to determine the water level using the DSM orthometric height. Both cross section lines were compared with tidal data for water level verification.

## 4. Results and Discussion

The following section presents the output of image acquisition by UAV comprising an image geo-referencing assessment of the UAV photogrammetry and the vertical accuracy of the DSM. The vertical accuracy of the DSM was compared with the water level between high and low tides, and both tide levels were validated with tidal data. The last part of the discussion is about the relevance of UAV–GNSS measurement compared to LiDAR and Satellite Altimetry.

### 4.1. UAV Output

#### 4.1.1. Results of SFM Image Processing

**Figure 8:**
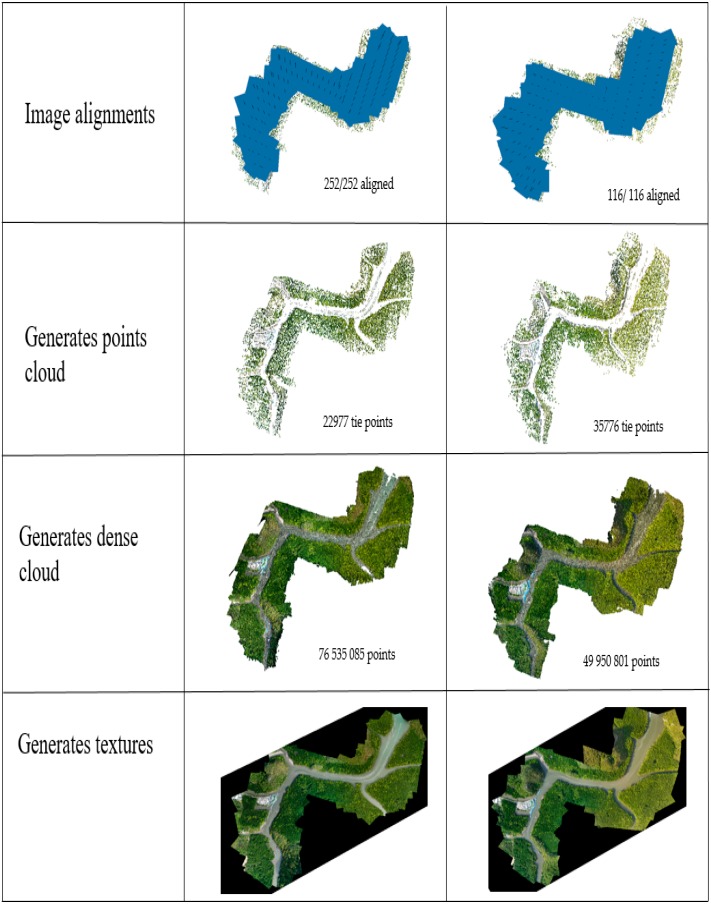
Image photogrammetric results at different stages for Epoch 1 (**left**) and Epoch 2 (**right**).

The results of SFM image processing include image alignments, generation of a point cloud, and dense cloud creation, as well as mesh and texture establishment. Figure 8 shows the results of each photogrammetric process at Epochs 1 and 2.

#### 4.1.2. Image Geo-Referencing Assessment

Table 3 displays the assessment of camera parameters for Epochs 1 and 2 based on focal length (F), principal point coordinates (Cx and Cy), radial distortion polynomial coefficients (K1, K2, and k3), and tangential distortion coefficients (P1, P2, P3, and P4). Both epochs show different values. Epoch 1 shows lower values of all parameters compared to Epoch 2, which shows significant changes.

**Figure 9:**
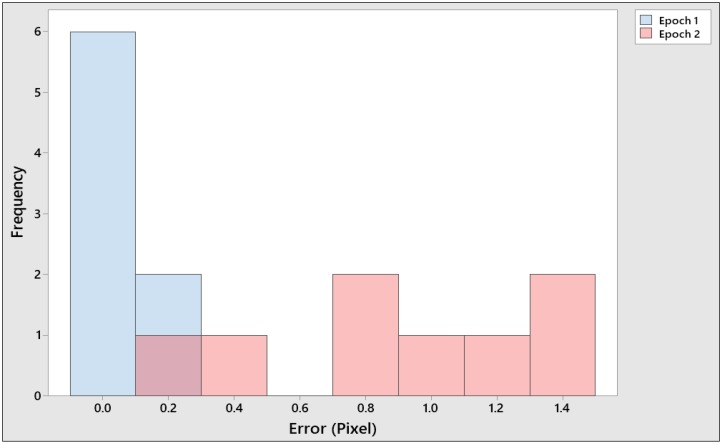
Histogram of the image geo-referencing error; (**A**) Error in pixels for Epoch 1; (**B**) Error in pixels for Epoch 2.

Table 4 contains the results for both epochs. In all cases, over ten thousand tie points were obtained. The table also shows over one million dense cloud points for both epochs. The final value of the root-mean-square error (RMSE) in pixels is shown. In all cases, the RMSE was below 1 pixel. Epoch 2 shows a higher value of RMSE than Epoch 1, as shown in Table 4. After the use of GCPs in image processing, the orientation procedure was completed, and the pixel error is shown in a histogram (Figure 9). Based on Figure 9, the pixel error was in the range 0.00 to 0.2 for Epoch 1, while for Epoch 2, the pixel error ranged from 0.2 to 1.4; this shows that the pixel error for Epoch 2 was higher than that for Epoch 1. Residuals for the GCPs for Epoch 1 were also calculated to be 0.196 (m), while the total error was 0.890 (pixels). For Epoch 2, the residual for GCPs was 0.010 (m), while the total error was 0.069 (pixels). This process also included the camera self-calibration that gave the results in Table 3. The focal length for both epochs was 8.8 mm, while the pixel size for both epoch was 2.61 × 2.61 µm.

### 4.2. Vertical Accuracy of the DSM

#### 4.2.1. Comparison of Water Level between High and Low Tide

**Figure 10:**
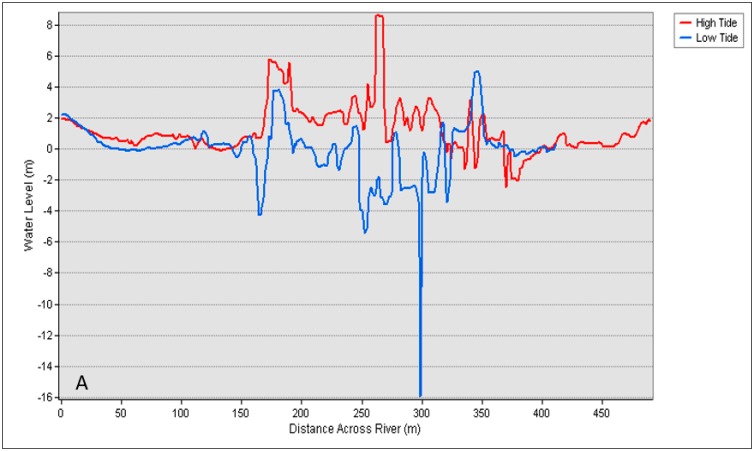

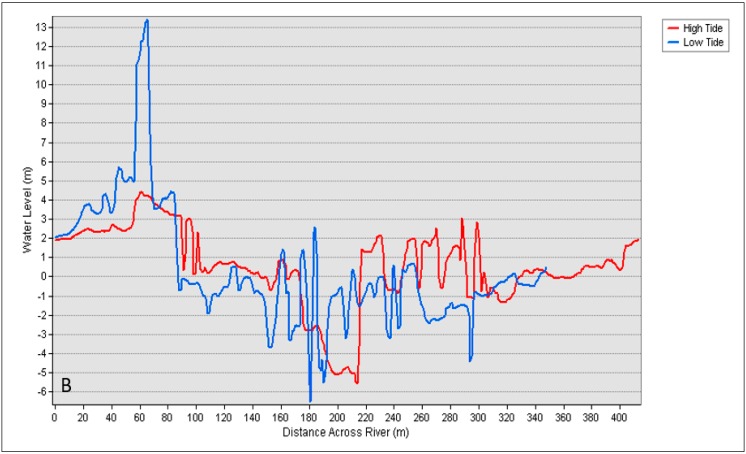
Water level profile (**A**) at cross Section 1; (**B**) at cross section 2.

To compare the water level between high and low tide, the DSM in Figure 7 was used. The line profile shown in Figure 10 visualizes the difference in water level during different tidal phases and also shows the condition of the water surface. The two cross section lines established in Figure 7C,D illustrate the noise or disturbance on the water surface affected by many factors such as the movement of boats or natural factors such as waves. Significant movement on the water surface only appeared in the middle of the line profile, which signified the middle of the river. It was assumed that boat or vessel movement disturbed the water surface, which caused the surface of the water to appear as in Figure 10.

**Figure 11:**
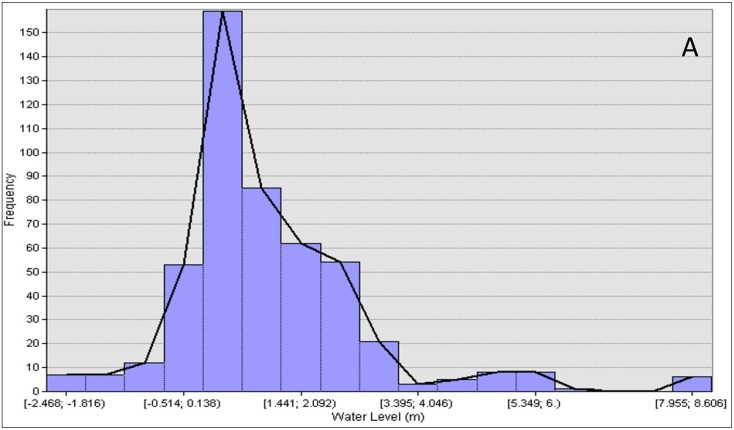

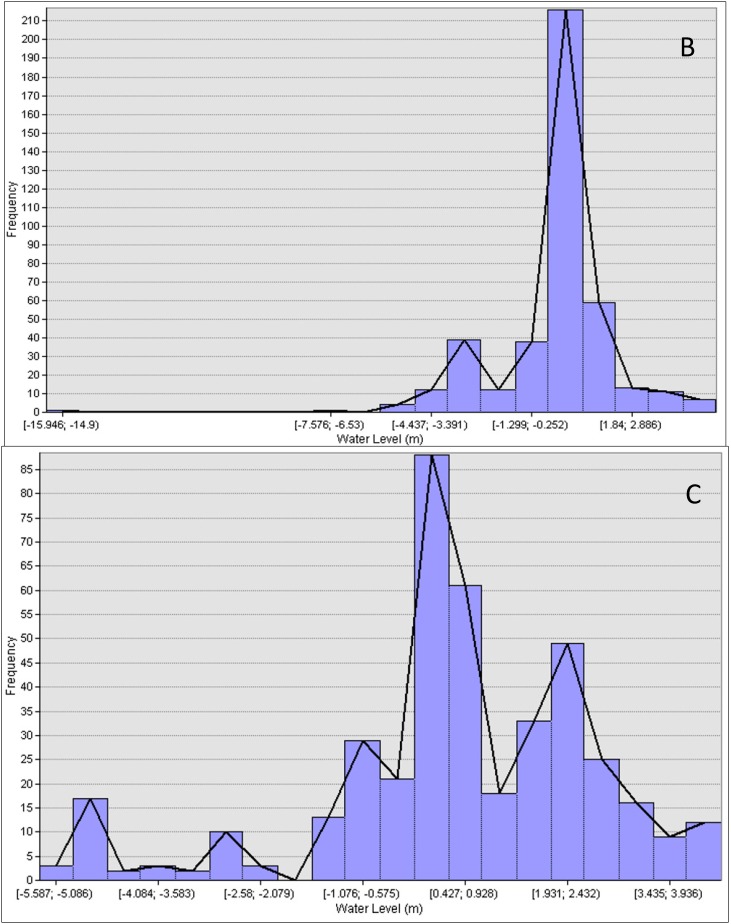

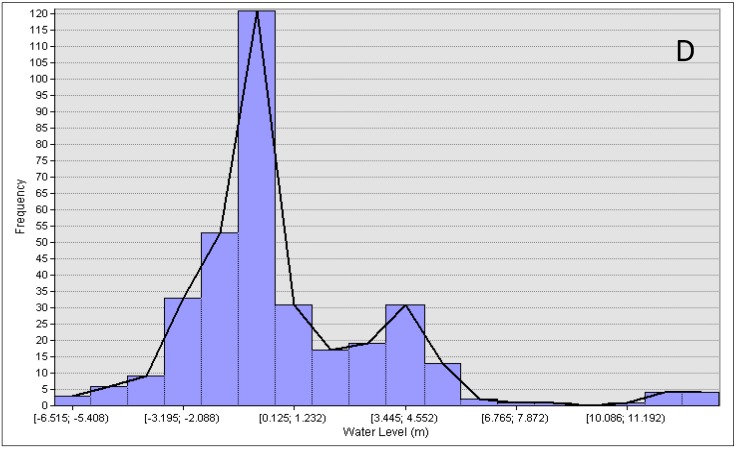
Histogram of water level against frequency; (**A**) Cross section 1 (high tide); (**B**) Cross section 1 (low tide); (**C**) Cross section 2 (high tide); (**D**) Cross section 2 (low tide).

According to Figure 10A, between 150 and 350 m across the river, the river profile showed noise or disturbance on the water surface because of interference by a vessel or boat. A similar situation happened in Figure 10B, which also displayed disturbance on the water surface. However, a significant difference between high and low tide still appeared at the edge of the line. Figure 11 shows a histogram of water level against frequency which displays the average of the water level at high and low tide. During high tide at cross section 1, the water level ranged from −0.514 to 3.395 m in Figure 11A,B, while at low tide, the water level ranged from −4.437 to 2.2886 m. Meanwhile, at cross section 2 in Figure 11C,D, the water ranged from −1.076 to 2.432 m during high tide, while at low tide, the water level ranged from −3.195 to 1.232 m.

#### 4.2.2. Comparison between DSM Orthometrics and Tidal Data

**Figure 12:**
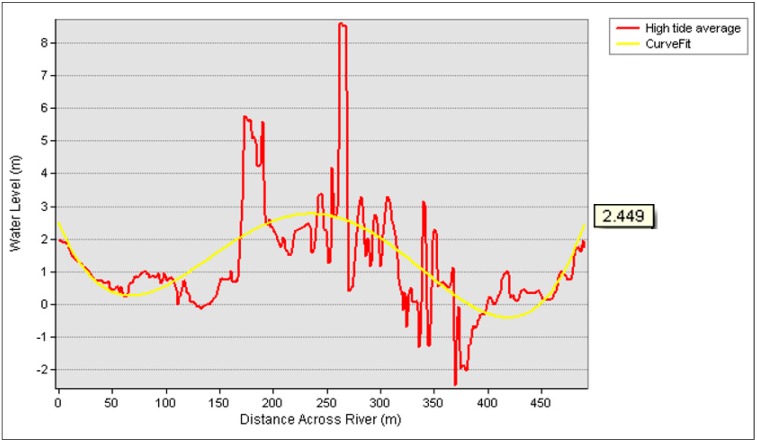
High tide average against tidal value at cross section 1.

To compare the water level extracted from the DSM orthometric height with tidal data, cross sections 1 and 2 were also used. The line profile in Figure 12 represents the water level surface during high tide, while tidal data were plotted in a curve fit line to represent the average tidal level. 

**Figure 13:**
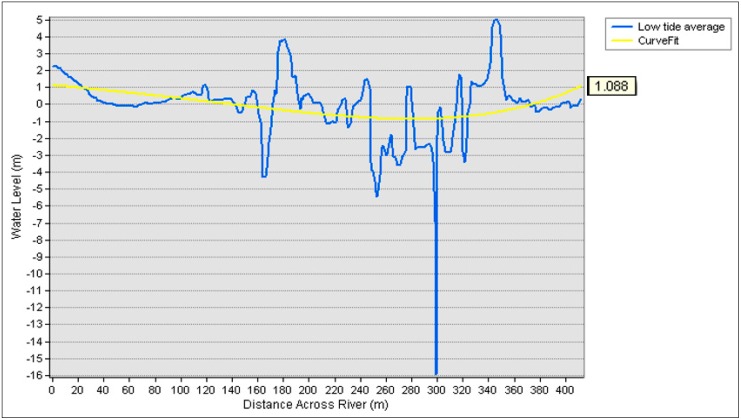
Low tide average against tidal value at cross section 1.

Figure 12 represents the water level at cross section 1 from the DSM against tidal values at high tide (from 13.58 p.m. to 16.16 p.m.) which resulted in 2.449 m as the average tidal value. Meanwhile, Figure 13 illustrates the water level at cross section 1 from the DSM against tidal values at low tide (from 10.56 a.m. to 12.29 p.m.) which resulted in 1.088 m as the average tidal value. A curve fit was established to represent the average tidal data level (Table 1) in the tidal range in either high or low tide. The residual of the water level from the DSM orthometric height and tidal data for high tide was 0.126 m (12.6 cm), while that for low tide was 0.345 m (34.5 cm).

#### 4.2.3. Relevance of UAV–GNSS Measurement Compared to LiDAR and Satellite Altimetry

The vertical accuracy of a UAV with GNSS positioning data shown in the previous section was 0.126 m (12.6 cm) for high tide, while for low tide it was 0.345 m (34.5 cm). According to DJI [32], the vertical accuracy for this model is ±0.5 m (with GNSS positioning), and the results show acceptable accuracy. This vertical accuracy was attained using the DSM orthometric height against tidal data, which were considered the reference data. This combination with GNSS positioning data provides UAV photogrammetry with high accuracy for vertical data, almost comparable with LiDAR and TLS.

In terms of data acquisition, UAV and GNSS combined measurement is flexible since the possibility to encounter bad weather can be avoided. During flight planning, the pilot is able to choose the time for data collection by considering weather problems such as rain intensity, wind, and other meteorological problems that could affect the flights. Data acquisition using a satellite platform has limitations since the user fully relies on satellite conditions to get good data, and the user cannot control the data according to the needs of their study. If the data do not satisfy the user, new data must be collected until the needs of their research are met. For LiDAR data, the cost per flight is expensive, especially when using rented instrumentation. The flight should be planned wisely to avoid any error because the cost for re-collection is expensive.

The Kilim River is surrounded by mangrove forest and experiences tidal phenomena, making it difficult to access for field measurement of water level changes. Using LiDAR is expensive for a 0.689 km^2^ area as it would require more than two flights and the setup is very complicated. Its cheapness and similar accuracy when compared with LiDAR and TLS encourage the usage of UAV–GNSS for the measurement of water level changes at Kilim River. The SFM algorithm in Agisoft Photoscan software allows the generation of high-accuracy DSMs and orthomosaics which are able to identify water level changes at different tidal phases.

## 5. Conclusions

This study showed the potential of UAV photogrammetry and GNSS vertical data for the generation of DSMs to identify water level changes at different tidal phases. This method allowed for water level measurements over an intertidal area of 0.689 km^2^ along the Kilim River with dense spatial resolution (5.2 cm at Epoch 1 and 6.17 cm at Epoch 2). On 20 December 2017, two epochs of image acquisition were acquired at the study area during low and high tide. In this study, eight GCPs were used for geo-referencing with total errors of 0.069 (pixels) for Epoch 1 and 0.890 (pixels) for Epoch 2. The SFM algorithm, implemented through software, was employed to generate a DSM and orthomosaics; this included several processes such as image alignment, camera optimization, building a point cloud and dense cloud, and building a mesh and texture model.

Using the DSM, cross-sectional lines were used to extract the profiles of water level surfaces for comparison between high and low tide, as well as for comparison with tidal data. Cross section lines were established across the river at a strategic location. During high tide at cross section 1, the water level ranged from −0.514 to 3.395 m, while at low tide, the water level ranged from −4.437 to 2.2886 m. Meanwhile, at cross section 2, the water ranged from −1.076 to 3.936 m during high tide, while at low tide, the water level ranged from −3.195 to 1.232 m. The results coincide with the conditions of the water level, which increases during high tide and decreases during low tide with such particular values. By comparison between the DSM and tidal data, the residual of the water level was 0.126 m (12.6 cm) for high tide, while for low tide, it was 0.345 m (34.5 cm). This value verifies that the UAV vertical accuracy is within ±0.50 m (50 cm).

In conclusion, the combination of UAV and GNSS vertical data is vital to identifying water level changes during different tidal phases at the Kilim River. Each type of data plays its role in identifying the water level in the 3D profile with high vertical accuracy supported by GNSS data. In addition, the availability of additional data, for example, MyGeoid data from DSMM, is highly appreciated since these data are valuable to calculating the orthometric height. Integrating aerial photogrammetry from a UAV platform with field measurement data could verify the accuracy of both approaches and the relationship between them. The results show only a slight bias for vertical accuracy (within ±0.5 m) between the DSM water level height and tidal data. Perhaps this study could benefit future research in exploring validation methods of water level surface measurements using UAV against tidal data from tide gauges.

## Figures and Tables

**Table 1:** Tidal conditions with respect to marine chart data for both epochs (20 December 2017).

Epoch	Period	Hour	Tidal Reading per Hour (m)	Tidal Range (m)	Average Tidal Level (m)
1	10.56 a.m.–12.29 p.m.	1000 1100 1200 1300	1.499 1.301 1.337 1.593	0.292	1.433
2	13.58 p.m.–16.16 p.m.	1300 1400 1500 1600	1.593 2.056 2.624 3.136	1.872	2.575

**Table 2:** Details of GCPs at the study area.

GCP	Longitude (°)	Latitude (°)	Ellipsoid Height h (m)	Geoid Height N (m)	Orthometric Height H (m)
1	99.8583050	6.404147	−13.542	−15.476	1.934
2	99.858142	6.403674	−13.506	−15.476	1.97
3	99.858608	6.401791	−12.892	−15.47	2.578
4	99.858971	6.405156	−13.395	−15.476	2.081
5	99.859376	6.40732	−13.495	−15.479	1.984
6	99.85912	6.4077478	−13.693	−15.481	1.788
7	99.862651	6.40594	−13.451	−15.464	2.013
8	99.86401	6.406795	−13.344	−15.46	2.116

**Table 3:** Camera parameters for both epochs of flight.

Camera Parameters	Epoch 1	Epoch 2
F	3183.75	4002.38
Cx	−2.86094	−27.3149
Cy	25.4618	10.0872
B1	−37.2172	121.949
B2	−1.73538	82.3614
K1	0.00250922	0.0102225
K2	−0.0126191	−0.0258016
K3	0.00931268	0.037059
P1	−7.4061 × 10^−5^	−9.27189 × 10^−8^
P2	7.29483 × 10^−5^	5.55727 × 10^−8^
P3	22.0224	1724.83
P4	−19.0707	228.112

**Table 4:** Assessment of the image geo-referencing error from GCPs for both epochs.

Epoch	GSD (cm)	No. of Photos Used	Tie Points	Dense Cloud Points	Error (pixel)
1	5.2	252	22,977	76,535,085	0.069
2	6.17	116	35,776	49,950,801	0.890
